# The effects of low-intensity narrow-band blue-light treatment compared to bright white-light treatment in sub-syndromal seasonal affective disorder

**DOI:** 10.1186/s12888-016-0729-5

**Published:** 2016-02-18

**Authors:** Ybe Meesters, Wim H. Winthorst, Wianne B. Duijzer, Vanja Hommes

**Affiliations:** University of Groningen, University Medical Center Groningen, University Center for Psychiatry, PO Box 30001, Groningen, 9700 RB The Netherlands; Philips Consumer Lifestyle Drachten, Drachten, The Netherlands

**Keywords:** sub-SAD, Light treatment, Narrow-band blue light

## Abstract

**Background:**

The discovery of a novel photoreceptor in the retinal ganglion cells with a highest sensitivity of 470-490 nm blue light has led to research on the effects of short-wavelength light in humans. Several studies have explored the efficacy of monochromatic blue or blue-enriched light in the treatment of SAD. In this study, a comparison has been made between the effects of broad-wavelength light without ultraviolet (UV) wavelengths compared to narrow-band blue light in the treatment of sub-syndromal seasonal affective disorder (Sub-SAD).

**Method:**

In a 15-day design, 48 participants suffering from Sub-SAD completed 20-minute sessions of light treatment on five consecutive days.

22 participants were given bright white-light treatment (BLT, broad-wavelength light without UV 10 000 lux, irradiance 31.7 Watt/m^2^) and 26 participants received narrow-band blue light (BLUE, 100 lux, irradiance 1.0 Watt/m^2^). All participants completed daily and weekly questionnaires concerning mood, activation, sleep quality, sleepiness and energy. Also, mood and energy levels were assessed by means of the SIGH-SAD, the primary outcome measure.

**Results:**

On day 15, SIGH-SAD ratings were significantly lower than on day 1 (BLT 54.8 %, effect size 1.7 and BLUE 50.7 %, effect size 1.9). No statistically significant differences were found on the main outcome measures.

**Conclusion:**

Light treatment is an effective treatment for Sub-SAD. The use of narrow-band blue-light treatment is equally effective as bright white-light treatment.

**Trial registration:**

This study was registered in the Dutch Trial Register (Nederlands Trial Register TC = 4342) (20-12-2013).

## Background

Seasonal Affective Disorder (SAD), winter type, is a well-studied syndrome, characterized by almost yearly recurring depressive episodes in autumn/winter alternating with symptom free episodes in spring/summer. The syndrome was recognized in the early eighties of the previous century and has been included in consecutive editions of the Diagnostic and Statistical Manual of Mental Disorders [1]. This classification system describes the syndrome as a seasonal pattern of major depressive disorder or alternatively as a bipolar I or bipolar II disorder. SAD is a serious affective disorder, and those suffering from it often need professional help. Exposure to bright light is the treatment of choice for patients suffering from SAD winter type [2–4]. An increasing number of studies have shown the positive effects of light treatment on this type of seasonal depression.

Apart from people suffering from SAD, there are those suffering from less severe complaints in wintertime. The main difference is that, according to the diagnostic criteria of DSM, the latter fail to meet the number of complaints required for a DSM diagnosis. Because the diagnostic criteria of SAD are not fulfilled, these complaints are known as sub-syndromal SAD (sub-SAD). Sub-SAD can be considered to be part of a continuum of seasonality between no complaints at all and severely depressed.

### Sub-syndromal SAD

People suffering from sub-SAD in wintertime generally experience hypersomnia, a lack of energy, a craving for carbohydrates and weight gain, or a decreased interest in socializing. These symptoms sometimes are accompanied by a lowered mood, but not by an actual depression.

Kasper et al. [5, 6] have described the criteria of sub-SAD. These include a regular pattern of seasonal (winter) problems (in at least two consecutive winters for a minimum period of 4 weeks), such as decreased energy levels, less efficiency at work, decreased interest in socializing, changed eating habits (eating more carbohydrates and weight gain) and changed sleep patterns (more sleep). Subjects regard these difficulties as normal and do not see them as the symptoms of an illness. They do not seek professional help, nor do others suggest they do so. These difficulties are not recognized by people outside the subjects’ inner social circle and are easily attributed to “being overworked” or “having the flu”. The symptoms are not disturbing subjects’ lives in any major degree. Subjects have no history of major depression, nor do they suffer from any physical illness.

Although sub-SAD complaints are less severe than those of SAD, this does not imply that sub-SAD is completely harmless. Lack of energy or hypersomnia can, among other things, lead to social dysfunctioning, frustrate educational opportunities, or lead to problems in relationships or work-related problems in wintertime. Several studies have described positive effects of light in treating sub-SAD [7–10]*.*

Kasper et al. also formulated sub-SAD criteria based on the Seasonal Pattern Assessment Questionnaire (SPAQ, [11]). These are often used in epidemiological studies [12] to discriminate between SAD and sub-SAD. Among these criteria, the score on a subscale of the SPAQ, the Global Seasonality Score (GSS), is used as a cut-off point. The results of these studies show that the prevalence of sub-SAD is about 2-4 times higher than the prevalence of SAD [13–15].

However, the use of the SPAQ as a discriminator between SAD and sub-SAD has been criticized [16, 17]: a high score on the GSS does not make a person depressed by definition. In view of this, in the present study, we used the Mini-International Neuropsychiatric Interview (MINI, [18]) for diagnosing/excluding depression. Potential participants who fulfilled the criteria of a mood disorder according to the MINI were excluded from the design. The Structural Interview Guide for the Hamilton Depression Rating Scale-Seasonal Affective Disorder (SIGH-SAD, [19]) was used for measuring the severity of the depressive symptoms.

### Blue light

The discovery of a novel photoreceptor in the retinal ganglion cells [20–23] with a maximum sensitivity of 470–490 nm to blue light has led to research on the effects of short-wavelength light on humans. These non-image forming (NIF) photoreceptors play a role in regulating the biological clock [24–26], but also project directly to several other areas of the brain [22, 27].

Several studies have explored the efficacy of monochromatic blue or blue-enriched light in the treatment of SAD. When more blue light was added to the spectrum of a bright-light treatment lamp (10 000 lux, Tcc = 17 000 K) the response was similar to that after exposure to bright white-light therapy (BLT, 10 000 lux, Tcc = 5 000 K) [28]. A possible explanation of this result was thought to be the saturation of the ocular receptors due to very high illuminance levels. In order to examine this, another study using the same design has compared moderate-intensity blue-enriched light (750 lux, Tcc = 17 000 K) with BLT (10 000 lux, Tcc = 5 000 K) in the treatment of SAD. This study did not show any differences in the therapeutic responses between the two light conditions [29] either.

With the invention of blue light-emitting diodes, narrow-band light sources fitting the maximum sensitivity of the NIF photoreceptors became readily available. Studies were undertaken to explore the use of LED technology in the treatment of seasonal complaints comparing the effects of light of different wavelengths. The therapeutic responses to blue light were found to be superior to those of red light [30, 31]. The effects of blue-enriched white LED light were found to be superior to placebo (deactivated ion-generator) [32] but similar to the effects of blue light [33]. In a study comparing the effects of low-intensity narrow-band blue light (BLUE) to the effects of high-intensity BLT, no differences in therapeutic outcome were found [34].

Although the effects of both light treatment and blue or blue-enriched light specifically have been studied repeatedly in SAD populations, only a small number of studies investigating the effects of light treatment in sub-SAD is available.

This is the first study exploring the effects of blue light on people with sub-SAD. As a control we used bright white-light therapy. We assessed effects on mood, energy, different aspects of activation, sleepiness, and sleep quality in the two conditions and looked for possible differences related to age and gender. In a previous study of an SAD population [28] no difference in efficacy was found between daily 20- and 30-minute sessions of exposure to bright light. In this study the effects of white and blue light in a 20-minute treatment have been compared for the first time. We hypothesize that both treatments are effective and that the therapeutic effects of BLUE are larger than those of BLT.

The human lens yellows with age, and yellow lenses can filter out short-wavelength light. Therefore older participants may profit less from the blue-light condition. In this study, the results of older participants have also been compared to those of younger participants.

## Methods

In the winters of 2010-2011 and 2011-2012 (October 1st to February 10th) subjects were recruited mainly by means of advertisements in regional papers, websites and small news items in magazines. Potential participants were roughly screened by means of a short phone survey. After that written information was sent to them and they were invited for an interview at the SAD outpatient clinic of the University Center for Psychiatry in Groningen. Participants in the screening interview were assessed by means of a standardized structured interview, the Mini-International Neuropsychiatric Interview (MINI, [18]). Subjects meeting the criteria of depression or any other psychiatric diagnosis from the DSM-IV-TR [1] were excluded from further screening.

The remaining subjects were assessed by means of the SIGH-SAD [19]. A SIGH-SAD score of ≥ 12 and < 18 was needed for inclusion in the design. Subjects with a score below 12 hardly differ from healthy people and were, therefore, excluded. In addition to the SIGH-SAD questionnaire subjects filled out the Seasonal Pattern Assessment Questionnaire (SPAQ, 7). The Global Seasonality Score (GSS) had to be ≥8 for a subject to be included in the study, but the seasonal complaints also had to be mild enough so as not to interfere with the subjects’ daily lives.

The 15-day protocol started at the latest 7 days after the screening interview. Participants underwent a 20-minute light treatment at home on five consecutive working days (days 4–8), which had to be completed by 8.20 a.m. This period of 5 days was based on SAD studies that have shown such a period to be effective [35, 36]. Subjects were asked to complete daily questionnaires concerning mood, sleepiness and sleep quality throughout the protocol, and mood and fatigue questionnaires on a weekly basis. The subjects visited the clinic on days 1, 8, and 15. The first 3 days before light treatment served as a baseline. A number of studies have shown that the response after light treatment still increases after termination of the light exposure [28, 29, 34, 35, 37]. Therefore, the assessment procedures were continued for 7 more days after the light exposure had ended.

### Light treatment

Subjects were randomised (controlling for age and gender) to one of the two treatment modalities: either to the low-illuminance blue-light therapy (BLUE) group or to the bright white-light therapy (BLT) group. The interviewers were unaware of the treatment subjects were assigned to. Before starting the interview, participants were asked not to give any information about the light condition to the interviewers.

BLUE light (goLITE HF3320, Philips Drachten, The Netherlands) characteristics were: peak LED wavelength 470 nm (full width half-maximum 25 nm), usage distance 50 cm positioned on a table top at 45 degrees sideways, vertical photopic illuminance 100 lux at eye position, irradiance 1.0 Watt/m^2^, equivalent melanopic illuminance 770 m-lux [38].

**Fig. 1:**
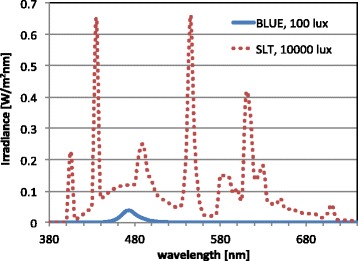
Spectral-power distributions of the lamps

The BLT was a white fluorescent lighting device (EnergyLight HF3319, Philips Drachten, The Netherlands), correlated color temperature 5 000 K, vertical photopic illuminance at 20 cm usage distance 10 000 lux, irradiance 31.7 Watt/m^2^; equivalent melanopic illuminance of 8620 m-lux [38]. Spectral distributions of the two treatment modalities are shown in Fig. 1.

The subjects were instructed to use the lamps as an addition to their normal room illumination. In that sense, the BLUE condition was actually blue-enriched white light. Assuming a 3 000 K TL spectrum, and an average background illumination of 250 lux, the effective equivalent melanopic illuminance in both cases increased by 115 m-lux, making the BLT condition one order of magnitude higher on melanopic illuminance.

### Assessment and procedure

We compared the two conditions on a weekly and daily basis to assess the same issues, using self-rating scales, and on a weekly basis using standardized structured interviews. Since all assessment procedures have their own shortcomings (weekly = retrospective; daily = assessment at the moment of the day; self-report: subjective assessment biased by the opinion of the participant; standardized structured interview: biased by the opinion of the interviewers) we used different assessment procedures to strengthen the assessment procedure.

Each of the two conditions started at day 1 (Friday) with a baseline measurement consisting of a SIGH-SAD interview (with interviewers blind to the light condition), the Beck Depression Inventory, second version (BDI-II-NL, [39]), a fatigue self-rating questionnaire (Short Fatigue Questionnaire, SFQ; [40]), and a questionnaire aiming to evaluate subjects’ expectations of the effects of light therapy. 5-point scale ratings were collected with the help of this latter questionnaire to check whether subjects expected to benefit from each treatment modality (white and blue light), whether they thought each was a logical treatment and whether they would recommend one or the other to a friend. They filled out this questionnaire before they had seen the light fixtures. The SIGH-SAD can be subdivided in a section containing the Hamilton Rating Scale of Depression (HRSD) assessing depressive symptoms, and a section assessing the atypical symptoms (ATYP) that are common in SAD, such as hypersomnia, a decreased need for socializing and carbohydrate craving. Subjects who met all inclusion criteria were randomly assigned to one of the two conditions, with gender and age distributed evenly over the groups.

The SIGH-SAD, the BDI-II-NL and SFQ were repeated at day 8 (after the 5th light session), and at day 15.

From day 1 onwards, participants filled out questionnaires on a daily basis before 8.00 a.m., 30 minutes after waking up at the latest, but before light had been applied. The mean scores on the questionnaires of the first four days were considered baseline.

These questionnaires dealt with mood, the Adjective Mood Scale (AMS, [41–43]); sleepiness, Karolinska Sleepiness Scale (KSS, [44]); and sleep quality, the Groninger Sleep Quality Scale (GSQS, [45, 46]). Also, four components of activation were measured, using the Activation Deactivation-Adjective Check List (AD-ACL, [47]): General Activation (GA; i.e. energetic, vigorous, full of pep, active and lively), Deactivation-Sleep (DS; i.e. sleepy, tired, drowsy, wide awake, and wakeful), High Activation (HA; i.e. jittery, intense, fearful, clutched-up, and tense), and General Deactivation (GD; i.e. placid, calm, at rest, still and quiet). Subjects were asked to describe their current feelings and to rate the 20 adjectives from the questionnaire on a 4-point scale.

### Statistics

Baseline differences between the scores of the two conditions on the SIGH-SAD, the BDI-II and the SFQ were tested by means of t-tests (continuous outcomes) and chi-square tests (dichotomous outcomes).

Effect sizes [48] were calculated for each condition. These effect sizes reflect the differences between baseline (day 1) and day 15. Results were based on the weekly assessments of the two conditions and were compared by means of repeated measures ANOVA. A responder was defined as a subject who improved by at least 50 %.

Linear Mixed Models were used to compare the two conditions on the basis of the daily self-rating questionnaires. An advantage of linear mixed models is that they allow the inclusion of random effects; i.e. parameters are allowed to vary across individuals. This may reveal heterogeneity in individual growth curves. We used models with time, condition, and the interaction between time and condition, with the baseline score as a covariate (baseline score = mean of the 4 pre-intervention scores). We fitted models with the baseline score and the 11 days after the start of the intervention as the repeated measures and allowed the slope to vary across individuals. Maximum likelihood estimation was used. We compared models with different variance-covariance matrices. Selection of the final model was based on the Bayesian Information Criterion (BIC; with lower values indicating better models). If the random effect for slope was found to be non-significant, this term was removed from the model (unless this resulted in a higher value of the BIC criterion). Performing residual diagnostics on the final models verified regression assumptions.

In a secondary analysis, the potential impact of gender and age on outcome were examined. To this end, the interaction time*condition*gender and time*condition*age were added to the models.

We also evaluated the correlations between the GSS scores and severity of complaints, both at baseline and throughout the study.

Analyses were carried out using SPSS 20. A two-tailed alpha level of 0.05 was used to determine statistical significance.

The research protocol was approved by the Medical Ethical Committee of the University Medical Center Groningen. All participants signed the informed consent form.

## Results

### Subjects

**Fig. 2:**
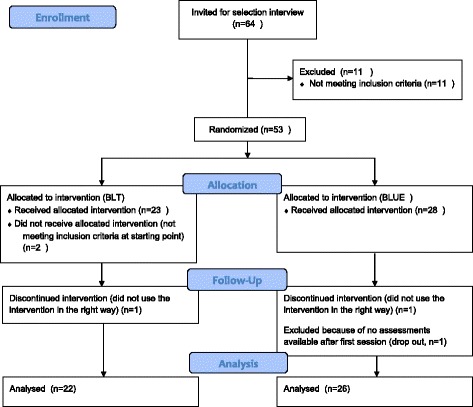
Flowchart inclusion of participants

Sixty-four potential participants were invited for a selection interview. Eleven were excluded on the basis of an unclear diagnosis, symptoms that were not severe enough or factors having an effect on mood. 53 participants started the study. Five other participants were excluded during the design. Two due to incorrect use of the lamp, one due to failure to complete the assessment procedures after Day 1, and two because the inclusion criteria did not match the scores at the moment of intake, which implies that inclusion would have been a mistake (Fig. 2).

Seven participants (3 in the BLT and 4 in the BLUE condition) scored >18 on the SIGH-SAD on day 1. In the screening interview their scores had been below 18. This difference was caused by their a-typical complaints, not by depressive symptoms. These subjects were, therefore, included in the analysis.

Ultimately, 22 participants (18 women, mean age 38.2 + 10.2, range 24–51 yr.; 4 men mean age 39.6 + 10.5, range 24–51 yr.) received white light and 26 participants (22 women, mean age 38.1 + 11.6, range 18–58 yr. and 4 men mean age 48.3 *+* 12.9, range 31–62 yr.) received blue light. If the subjects had been divided into two groups using Kasper’s SPAQ diagnostic criteria of the global seasonality scores (GSS), GSS < 11 and GSS > 10, baseline scores on SIGH-SAD were found to be the same in these groups.

### Weekly ratings

**Fig. 3:**
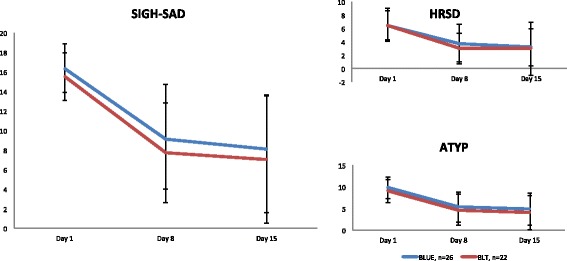
Scores on the SIGH-SAD (range 0-75). BLT = Bright Light Treatment, *n* = 22., BLUE = narrowband blue light treatment *n* = 26. SIGH-SAD = total score on the SIGH-SAD (24 item version, range 0-75); HRSD: sum score of the Hamilton Rating Scale for Depression items (17 item version, range 0-52) of the SIGH-SAD; A-TYP: sum score of the Atypical question items of the SIGH-SAD (range 0-23). Error Bars = standard deviation. For further explanation and abbreviations: see text

Both therapies were found to be highly effective in reducing the SIGH-SAD score and improving energy levels measured by the atypical symptom part (ATYP) of the SIGH-SAD (Fig. 3).

No statistically significant differences were found between light conditions on any of the weekly outcome measures. This also holds when the results are controlled for gender, age, severity of complaints (measured with the SIGH-SAD, HRSD, ATYP, or with the BDI-II, or SFQ), and expectations (measured with a self-rating questionnaire on day 1).

In both conditions the complaints assessed with the different instruments decreased during the 15-day period (Fig. 3 and Table 1): SIGH-SAD, 24 items, main effect ‘time’ F (2,45) = 53.9, *p* < 0.001), with no significant differences between conditions (main effect “condition” F (1,46) = 1.13, ns), nor over time between conditions (interaction effect “time*condition” F (2,45) = 0.06, ns). When dividing the SIGH-SAD in “typical” (HRSD) and “atypical” (ATYP) symptoms, the following is found: for HRSD (17-items Table 1) a main effect “time” F (2,45) = 22.04, ns; main effect “condition” F (1,46) = 0.35, ns; main effect “time*condition” F (2,45) = 0.28, ns and for ATYP (7 items, Table 1) a main effect “time” F (2,45) = 45.9, *p* = < 0.001; main effect “condition” F (1,46) = 1.09, ns; main effect “time*condition” F (2,45) = 0.05, ns. Based on the scores of the weekly assessed self-rating instruments the results for the BDI-II that were found had a main effect “time” F(2,45) = 35.13, *p* < 0.001; main effect “condition” F (1,46) = 0.11, ns; main effect “time*condition” F (2,45) = 1.77, ns. For the SFQ were found a main effect “time” F (2,45) = 16.77, *p* < 0.001; main effect “condition” F (1,46) = 0.20,ns; main effect “time*condition” F (2,45) = 0.1, ns.

**Table 1:** Weekly average scores (+ SD*)*

Instrument *(range)*	Condition	N	Day1 (SD)	Day 8 (SD)	Day 15 (SD)	Effect Size d	% Decrease	Responder N (in %)
SIGH-SAD *(0-75)*	BLT	22	15.5 (2.4)	7.7 (5.1)	7.0 (6.5)	1.7	54.8	13 (59)
	BLUE	26	16.3 (2.6)	9.1 (5.6)	8.1 (5.6)	1.9	50.7	16 (61.5)
HRSD *(0-52)*	BLT	22	6.5 (2.2)	3.0 (2.3)	3.0 (4.0)	1.1	53.8	13 (59)
	BLUE	26	6.5 (2.5)	3.8 (2.8)	3.2 (2.8)	1.2	50.7	17 (65)
ATYP *(0-23)*	BLT	22	9.0 (2.7)	4.7 (3.5)	4.0 (3.9)	1.5	55.6	15 (68)
	BLUE	26	9.8 (2.5)	5.3 (3.5)	4.9 (3.7)	1.6	50.0	14 (54)
BDI-II *(0-63)*	BLT	22	15.0 (4.3)	9.1 (5.1)	7.5 (5.8)	1.5	50.0	11 (50)
	BLUE	26	13.9 (4.8)	9.4 (5.7)	9.7 (7.5)	0.7	30.2	9 (35)
SFQ *(4-28)*	BLT	22	18.7 (3.3)	15.7 (3.9)	15.5 (3.5)	0.9	24.1	4 (18.1)
	BLUE	26	18.2 (3.4)	15.1 (3.5)	15.5 (4.1)	0.7	21.5	5 (19.2)

Cohen’s d effect size and response percentage from day 1 to day 15, as rated by the scale adapted for seasonal symptoms SIGH-SAD (24 items), the Hamilton Rating Scale for depression (HRSD, 17 items), and the atypical symptoms ATYP (7 items), the score on the Beck Depression Inventory-II (BDI-II) and the Short Fatigue Questionnaire (SFQ) for each condition. BLT = bright white light treatment; BLUE = narrow-band blue-light treatment. Responder = subject with an improvement of at least 50 %

In the absence of a clinical mood disorder, the atypical symptoms play a more prominent role in the seasonal difficulties as measured with the SIGH-SAD. At baseline, the subjects scored highest on fatigability and hypersomnia.

### Daily questionnaires

The results based on the scores of the daily self-rating questionnaires are shown in Table 2. One participant of the BLT condition was excluded from the analysis due to failure to fill out daily questionnaires during the baseline period and the majority of the following days. Consequently, in these analyses data of 47 subjects were used. The time effect was significant in all models. Both groups ended with equal scores on the daily questionnaires. For the KSS and General Deactivation subscale of the AD-ACL a significant effect of the interaction time*condition was found. Subjects receiving white light showed a larger decline in scores on the KSS and GD subscale of the AD-ACL compared to the blue light condition.

**Table 2:** Daily self-rating questionnaires

Outcome	Model	Estimate	P-value
Sleepiness (KSS)	Time	-0.139	.000
	condition	-0.561	.123
	time*condition	***0.089***	***.015***
	baseline	0.476	.000
Mood (AMS)	Time	-0.767	.007
	condition	-0.597	.738
	time*condition	0.335	.372
	baseline	0.788	.000
Sleep (GSQS)	Time	-0.116	.021
	condition	-0.263	.608
	time*condition	0.037	.583
	baseline	0.422	.002
Deactivation Sleep (AD-ACL)	Time	-0.188	.002
	condition	-0.390	.352
	time*condition	0.078	.330
	baseline	0.758	.000
General Activation (AD-ACL)	Time	-0.213	.003
	condition	-0.161	.744
	time*condition	0.100	.304
	baseline	0.746	.000
High Activation (AD-ACL)	Time	-0.032	.563
	condition	-0.473	.165
	time*condition	0.094	.216
	baseline	0.887	.000
General Deactivation (AD-ACL)	Time	-0.098	.029
	condition	-0.361	.225
	time*condition	***0.121***	***.047***
	baseline	0.901	.000

Results of regression analyses. Bold data reflects significant time*condition results

For the questionnaires concerning Mood, Sleep and the subscales Deactivation-Sleep, General Activation and High Activation of the AD-ACL the interaction time*condition was not significant.

We also examined the influence of gender and age on the outcomes. No interaction effects were found between gender or age on the one hand, and time or time*condition on the other. Adjustments for gender and age did not cause any substantial changes in the results either. Therefore, gender and age have not been included in the final models.

### SPAQ

The severity of the GSS score of the SPAQ is not related to the severity of baseline SIGH-SAD, HRSD or ATYP scores when two groups are created on the basis of a GSS cut-off score of 11 (Table 3). In the BLT group 8 out of 22 had a GSS score of 11 or higher (36.4 %), 1 subject had a GSS score of 17. In the BLUE group 7 out of 26 had a GSS score of 11 or higher (27.9 %). When comparing the two conditions (high vs. low GSS scores) based on the SIGH-SAD scores, we find the main effect ‘time’ F (2,45) = 50.55, *p* < 0.001, with no significant differences between conditions (main effect “condition” F (1,46) = 0.081, ns) or over time between conditions (interaction effect “time*condition” F (2,45) = 3.14, ns).

**Table 3:** Effects of high vs. low GSS score on primary outcome

	N	SIGH-SAD	SIGH-SAD	SIGH-SAD	HRSD	HRSD	HRSD	ATYP	ATYP	ATYP
		Day 1	Day 8	Day 15	Day 1	Day 8	Day 15	Day 1	Day 8	Day 15
GSS total < 11	33	15.64	9.1	7	6.6	3.6	2.6	9.1	5.5	4.4
GSS total > 10	11	16.67	7.1	8.9	6.3	3.1	4.1	10.4	4	4.8

SIGH-SAD scores, HRSD scores and Atypical scores(ATYP) on days 1, 8 and 15 in relation to SPAQ GSS scores. The subdivision of the scores on GSS items is based on median scores in the direction of the mean scores

### Side effects and evaluation

Participants spontaneously reported some side effects. 9 % of the participants in the BLT condition reported headaches, 9 % experienced headaches and nausea, and 9 % had headaches and felt hyper during the treatment. In the BLUE condition 8 % of the participants reported headaches, 4 % experienced headaches and nausea and another 4 % reported headaches and palpitations during the treatment. Also, in the BLUE condition, 4 % reported dry eyes and yet another 4 % reported diarrhoea during treatment. No statistically significant difference in the number of side effects between the conditions was found, though.

Participants seem to equally like the colours white or blue. Participants in the conditions would also like to get the same treatment the following year, as well as the remaining part of the season. The participants who received blue light were a little happier with the treatment than those receiving white light. However, there are no significant differences between the two conditions.

## Discussion

To the best of our knowledge, this is the first study looking at the effects of blue light on a population suffering from sub-SAD. We compared low-intensity blue-light treatment to the bright-white light treatment used in the treatment of sub-SAD.

Since this is a field study, there is no controlled for all factors that can contribute to the therapeutic outcome. We assume that those factors are equally distributed about the two conditions.

Both treatment conditions were highly effective in reducing symptoms of sub-SAD, as measured by weekly interviews and self-reported ratings. The effects of BLUE light treatment were comparable to those of the bright white-light treatment.

This is also the first time the effectiveness of only 20 minutes of daily light exposure has been studied. The SIGH-SAD analysis showed that both mood and atypical symptoms improved after one week of light intervention (see Fig. 3).

At baseline, the severity was mainly driven by atypical symptoms: lack of energy, fatigability, hypersomnia, and less so by mood disturbances. The most pronounced effect of light treatment was observed on the atypical symptoms of hypersomnia and fatigability. This is in line with observations of Rastad et al. [49]. Both HRSD and BDI-II ratings were relatively low at baseline: scores of 8 or lower on HRSD and 10 or lower on BDI-II are typical of healthy populations. This overall relatively low severity at baseline limits the range of improvement (and the percentage of responders defined by 50 % improvement of a score) compared to the effects of light treatment on SAD populations we observed in earlier studies [28, 34].

So far, no methodologically justified placebo condition is available for light treatment. In light research, various placebo-like conditions have been used, such as imaginary light [50]; invisible light [37], extra-ocular light [51], low-intensity light [29], or a placebo condition totally unrelated to light, such as a deactivated ion generator [32]. Responses to these ‘placebo’ conditions have varied from 36 to 46 %. Although the response rates in this study, in both conditions are higher (54.8 and 50.7 %) it cannot be ruled out with certainty that these effects are due to placebo effects.

In a number of epidemiological studies a subdivision between SAD and sub-SAD is made on the basis of the GSS scores of the SPAQ [8, 52]. In this study, there is no relation between the severity of the GSS scores and the severity of the seasonal difficulties as measured by the SIGH-SAD (Table 3). This is in line with the findings of Hardin et al. [17], Magnusson [16] and Terman [53]. Because of the overlap between subjects with sub-SAD and SAD who reached scores of GSS 11 or higher and report at least a moderate problem on the SPAQ it is argued that a GSS cut-off score of 17 is more realistic when making a distinction between these two groups [53, 54]. In our population, only 1 subject (2.1 %) reached a GSS score of 17, with a SIGH-SAD score of 21 and a HRSD score of 5 before treatment. These findings support our decision not to use SPAQ GSS scores as a criterion to subdivide groups of persons suffering from SAD and sub-SAD in this study.

Like previous studies comparing blue-enriched treatment modalities to BLT in the treatment of SAD [28, 29, 34], this study also shows no significant differences in the clinically relevant responses to light for the sub-SAD group. The difference in photopic illuminance of the two conditions is two orders of magnitude (10 000 lux vs 100 lux), whereas the difference in the equivalent melanopic illuminance [38] is about one order of magnitude. The equivalent melanopic illuminance of BLUE light treatment is of the same order of magnitude as melanopic illuminance of 1000 photopic lux, 5000 K white fluorescent light. No difference in effects on people with seasonal problems has been shown over a wide range of high light intensities. Similar saturation was reported for alerting effects of light [55], and for melatonin suppression [56], where responses were already at a maximum, and the same between 1000 and 10000 lux of white 4000 K fluorescent light. The finding that we observe the same magnitude of effects across a larger range of photopic illuminance, but similar ranges of melanopic illuminance as in the studies above supports the hypothesis that ipRGCs play a role in mediating the effects of light when treating SAD and sub-SAD.

The results of most daily questionnaires are in line with the results of the weekly ratings. Both groups ended with equal scores on these questionnaires. The only difference between the two conditions found was a quicker decrease of the scores of the KSS and GD subscale of the AD-ACL for subjects receiving white light compared to subjects receiving blue light. We have no explanation for these two exceptions. The questionnaires were filled out after waking up and before light treatment in the morning, except at day 1, when questionnaires were filled out at the clinic at the start of the programme later in the morning. When leaving out the data from day 1 from the calculations, this did not show a difference in the results, so possible circadian effects on the scores of day 1 did not influence the results.

Stuhlmiller [57] stated that the effects of the seasons on psychological changes are inconsistent and controversial and are influenced by the appreciation of cultural perception and adaptation. This study shows that exposure to artificial light is beneficial for sub-SAD sufferers. Mood, energy levels, fatigability and sleep improve. Therefore, we think it is helpful to recognise that seasonal difficulties are related to more than cultural perception and adaptation.

The results of this study are in line with the dual vulnerability hypothesis, [58], which suggests that there is a vulnerability for the influences of the seasons as well as a vulnerability for the development of a depression. The difference between SAD and sub-SAD may be that sub-SAD sufferers are less vulnerable to developing a depression but are vulnerable to the effects of the seasons.

The human lens yellows with age, and yellow lenses can filter out short-wavelength light. For this reason, we examined the effect of age in both conditions, but were especially interested in the effects of age in the BLUE condition. We did not find any age-related differences. There are several possible explanations for this: it may be that the light-intensity is still sufficiently high, causing the transmitted light to be sufficient for a therapeutic effect, or it may be that a different adaptation mechanism exists which compensates for lower transmission in the blue range. This latter explanation is supported by two studies looking at biological consequences of reduced blue-light exposure by changed lens transmittance [59, 60]. Giménez et al. [59] looked at the melatonin secretion of young subjects wearing orange lenses and found that after 2 weeks the response to light was the same as before they wore lenses. Najjar et al. [60] found decreased lens transmission in the blue range in older people, but without consequences for overall melatonin suppression. Based on our results with BLT or BLUE light conditions we cannot claim one or the other to be more effective for a certain age group, at least within the age range explored.

Although subjects were randomized to one of the intervention modalities, rather than being allowed to select the light colour of preference, the final evaluation does not reveal any significant differences in treatment appreciation. The side effects reported are in line with the earlier observed reactions to bright-light treatment.

## Conclusions

In this study, people with sub-SAD completed 5 days of light treatment at home by either bright white-light therapy or low intensity blue LED-light therapy. Their condition was assessed by blinded interviewers and by self-assessments on a weekly basis, as well as via daily questionnaires over 2 weeks. 20 minutes of exposure to morning light on 5 days resulted in improved mood and improved energy levels, reduced fatigability and hypersomnia symptoms, which are the most striking symptoms in this population. No significant difference in treatment efficacy was found, neither in any of the weekly measures, nor in the majority of daily assessments.

Symptoms of Sub-SAD can be reduced effectively with light treatment, with the use of narrow-band blue-light treatment being equally effective as bright white-light treatment.

## Abbreviations

AD-ACL: activation deactivation adjective checklist
GA: General Activation
DS: deactivation – sleep
HA: High Activation
GD: General Deactivation
AMS: Adjective Mood Scale
ATYP: atypical symptoms
BDI-II-NL: beck depression inventory, second version, Dutch version
BLT: bright white-light therapy
BLUE: blue light therapy
DSM: Diagnostic and Statistical Manual of Mental Disorders
GSQS: Groninger Sleep Quality Scale
HRSD: Hamilton Rating Scale for Depression
KSS: Karolinska Sleepiness Scale
MINI: Mini-International Neuropsychiatric Interview
NIF: non- image forming
SAD: Seasonal Affective Disorder
SFQ: Short Fatigue Questionnaire
SIGH-SAD: Structural Interview Guide for the Hamilton Depression Rating, Scale- Seasonal Affective Disorder version
SPAQ: Seasonal Pattern Assessment Questionnaire
GSS: Global Seasonality Score of the SPAQ
Sub-SAD: sub-syndromal seasonal affective disorder
